# Supplementary material for: Faecal microbiota transplantation protects against radiation‐induced toxicity
Source: EMBO Mol Med. 2017 Feb 27;9(4):448–61. doi: 10.15252/emmm.201606932 (PMC5376756; doi:10.15252/emmm.201606932)
Supplement: Supplementary file 1 — Appendix [file EMMM-9-448-s001.pdf]

Appendix “Faecal microbiota transplantation protects against radiation-induced toxicity”by Cui et al.

Appendix Figure Legends pp 2-3

Appendix Figures S1 to S7 pp 4-10

Appendix Table S1 pp 11

Appendix Figure Legends

Appendix Figure S1. The intestinal bacterial flora profile between male and female mice is different

A, B The Shannon diversity index and β diversity of intestinal bacteria in male (A) and female (B) mice were examined by 16S high throughput sequencing after 6 weeks of housing, n = 4 per group. Statistically significant differences are indicated: Student's *t*-test.

C, D The WBC counts in PB were assessed in saline-treated, ampicillin-treated, and streptomycin-treated male and female mice after 6.5 Gy TBI. n = 6. Significant differences are indicated shown relative to the saline-treated group: ***$P < 0.005$; Student's *t*-test.

E The difference in the intestinal bacterial structure at the genus level between male and female mice was assessed by 16S high throughput sequencing, n = 4.

Appendix Figure S2. FMT retains the gene expression profile of the small intestine after radiation

A Significantly enriched GO terms (top 30) induced by sex-mismatched FMT in male mice.

B Significantly enriched GO terms (top 30) induced by sex-mixed FMT in male mice.

C, D Heat map of genes significantly up- and down-regulated (red and green, respectively) in small intestine tissue from sex-mismatched (C) and sex-mixed (D) FMT male mice compared with those of the controls. Each row represents a single gene.

Appendix Figure S3. FMT retains the gene expression profile of the small intestine after radiation

A Significantly enriched GO terms (top 30) induced by sex-mismatched FMT in female mice.

B Significantly enriched GO terms (top 30) induced by sex-mixed FMT in female mice.

C, D Heat map of genes significantly up- and down-regulated (red and green, respectively) in small intestine tissue from sex-mismatched (C) and sex-mixed (D) FMT female mice compared with those of the controls. Each row represents a single gene.

Appendix Figure S4. Sex-matched FMT alters the lncRNA expression which related to immunity in male mice

Coding–non-coding gene co-expression network was analyzed in male mice. The green spots represent mRNAs and the yellow spots represent lncRNAs. Red lines represent a positive interaction between transcripts and blue lines represent a negative interaction.

Appendix Figure S5. Sex-matched FMT alters the lncRNA expression which related to metabolism in male mice

Coding–non-coding gene co-expression network was analyzed in male mice. The green spots represent mRNAs and the yellow spots represent lncRNAs. Red lines represent a positive interaction between transcripts and blue lines represent a negative interaction.

Appendix Figure S6. Sex-matched FMT alters the lncRNA expression which related to metabolism in female mice

Coding–non-coding gene co-expression network was analyzed in female mice. The green spots represent mRNAs and the yellow spots represent lncRNAs. Red lines represent a positive interaction between transcripts and blue lines represent a negative interaction.

Appendix Figure S7. Sex-matched FMT alters the lncRNA expression which related to immunity in female mice

Coding–non-coding gene co-expression network was analyzed in female mice. The green spots represent mRNAs and the yellow spots represent lncRNAs. Red lines represent a positive interaction between transcripts and blue lines represent a negative interaction.

Appendix Figure S1

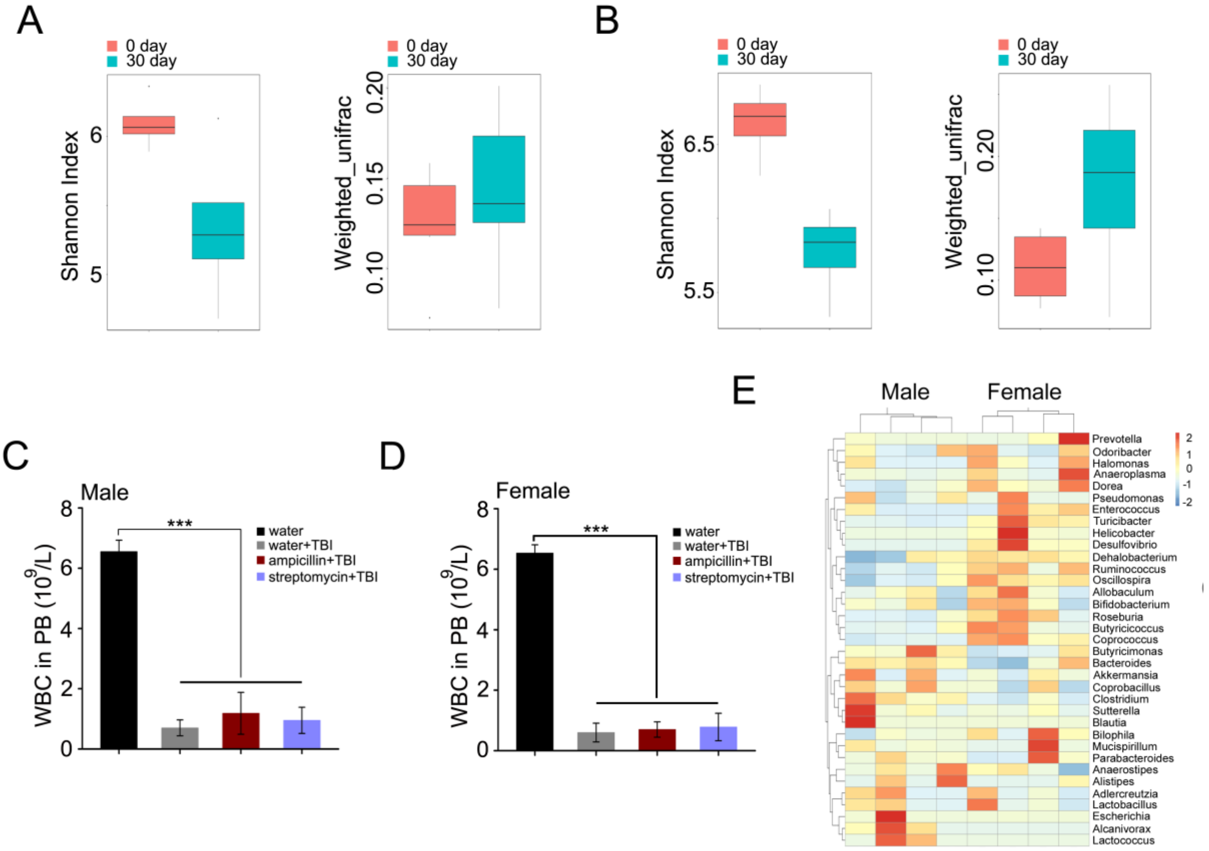

Appendix Figure S2

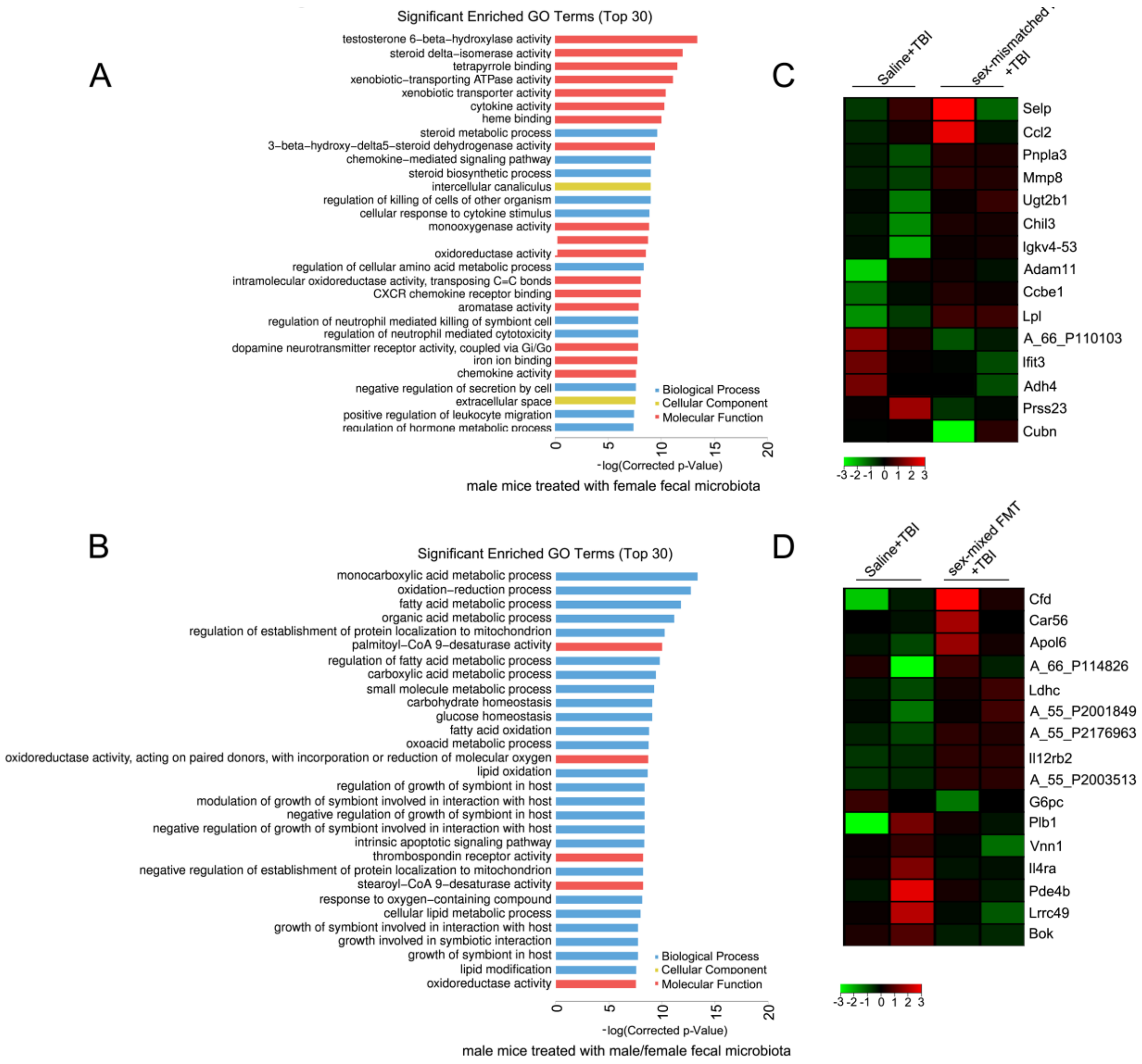

Appendix Figure S3

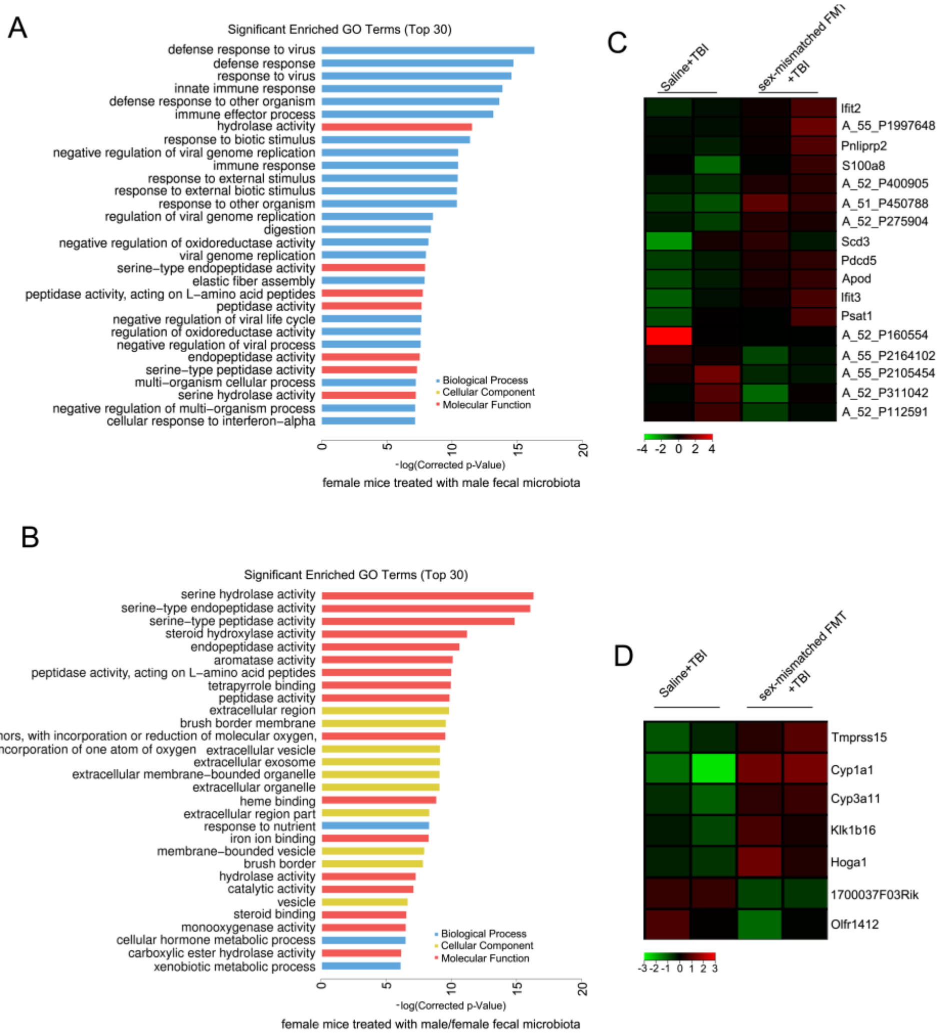

Appendix Figure S4

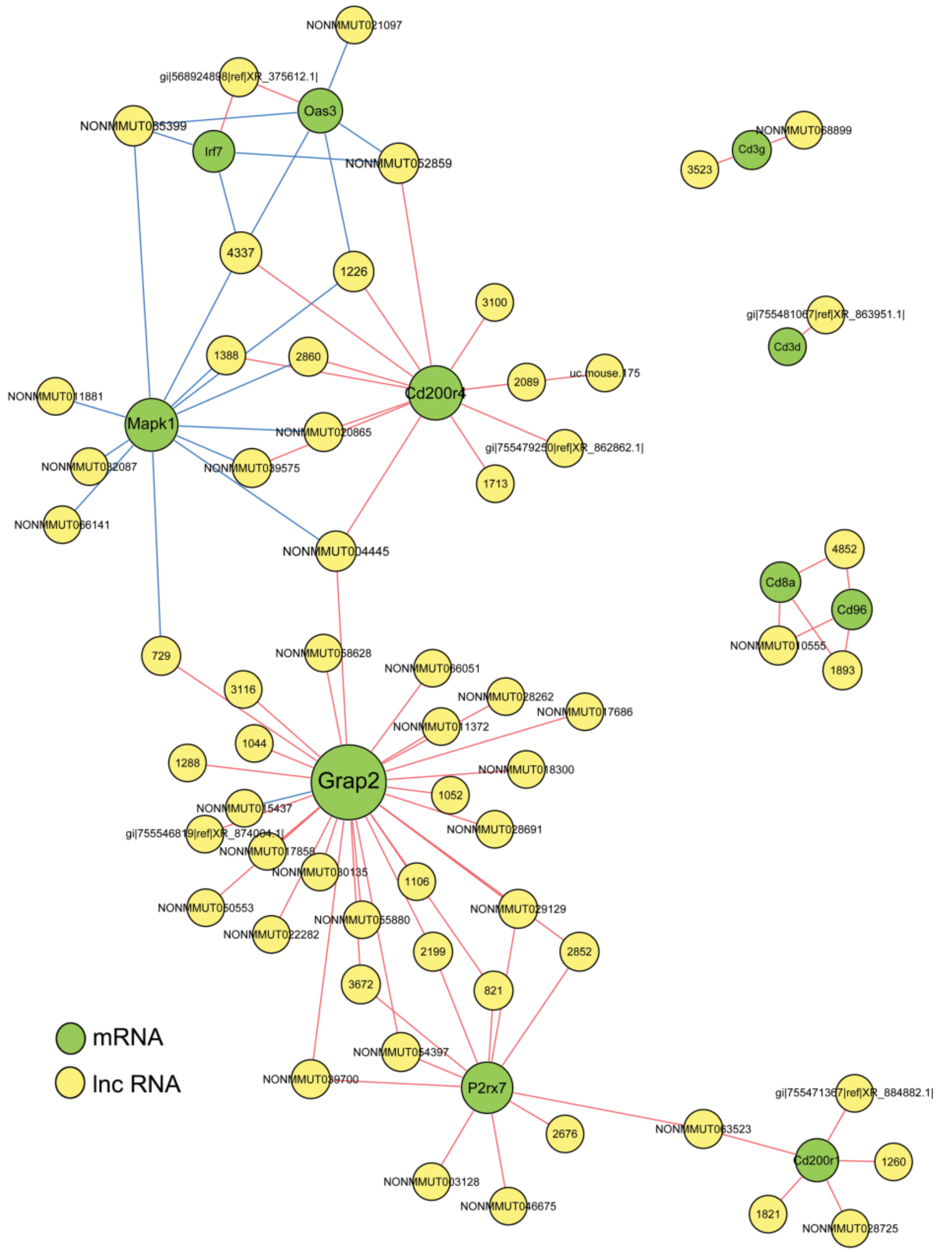

The alteration of lncRNA expression related to immunity in Male+TBI treated male mice

Appendix Figure S5

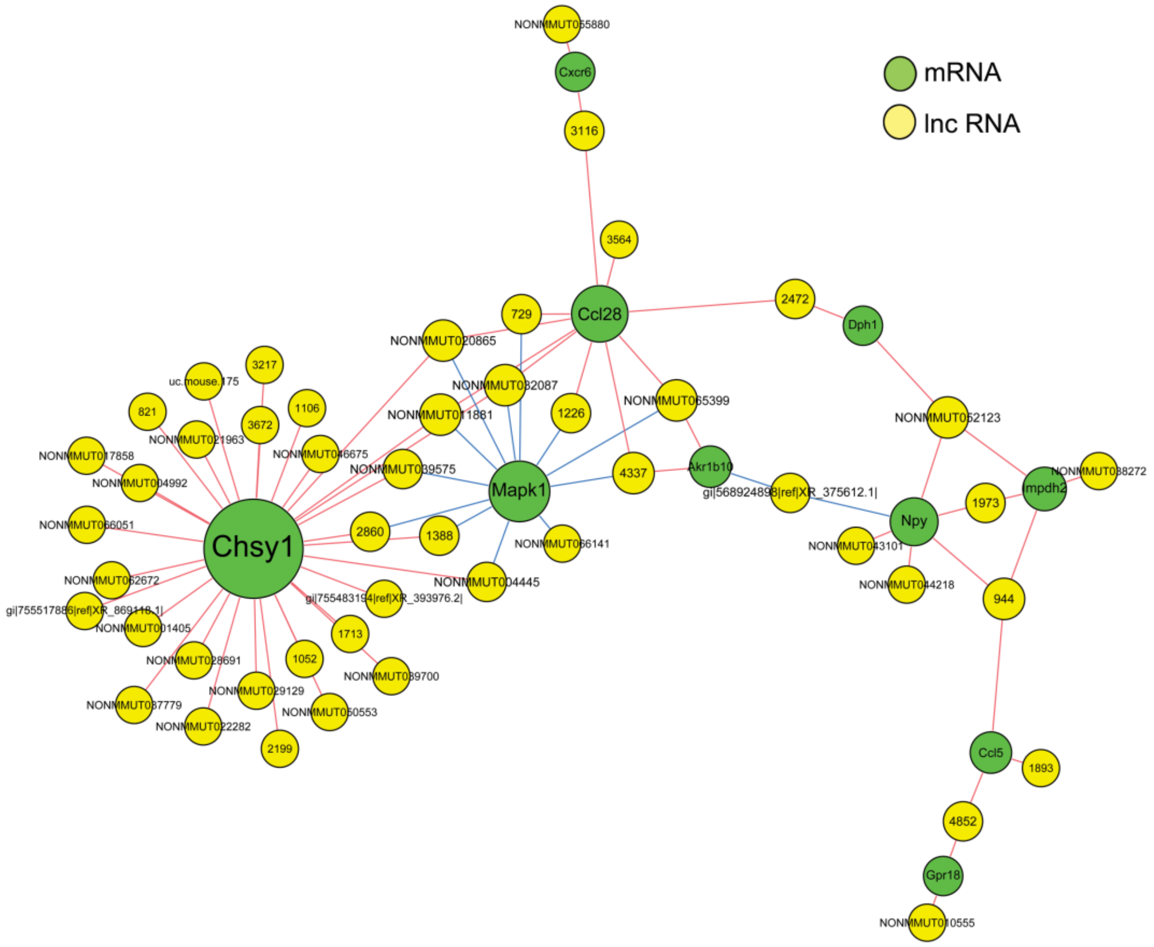

The alteration of lncRNA expression related to metabolism in Male+TBI treated male mice

Appendix Figure S6

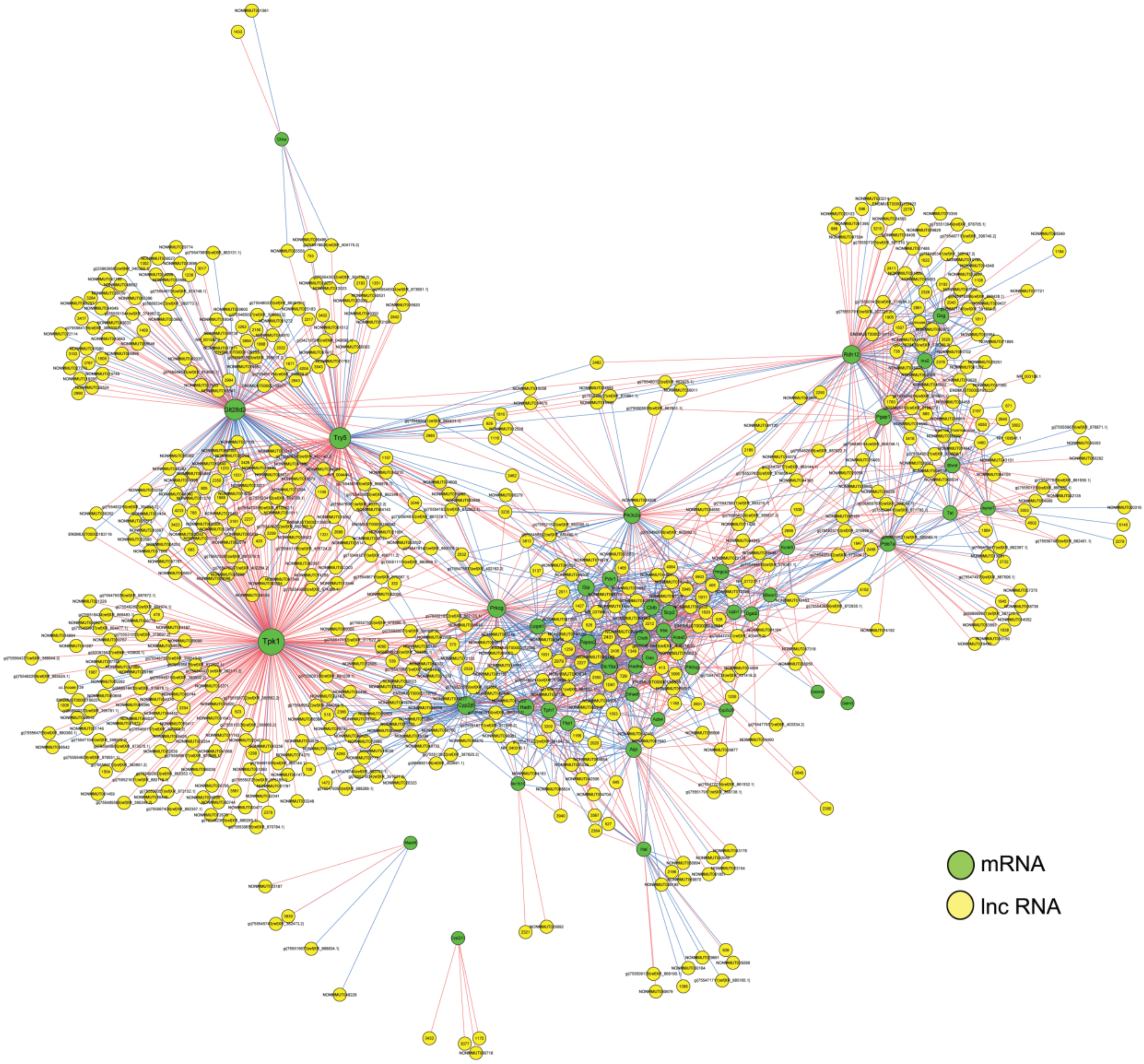

The alteration of lncRNA expression related to metabolism in Female+TBI treated female mice

Appendix Figure S7

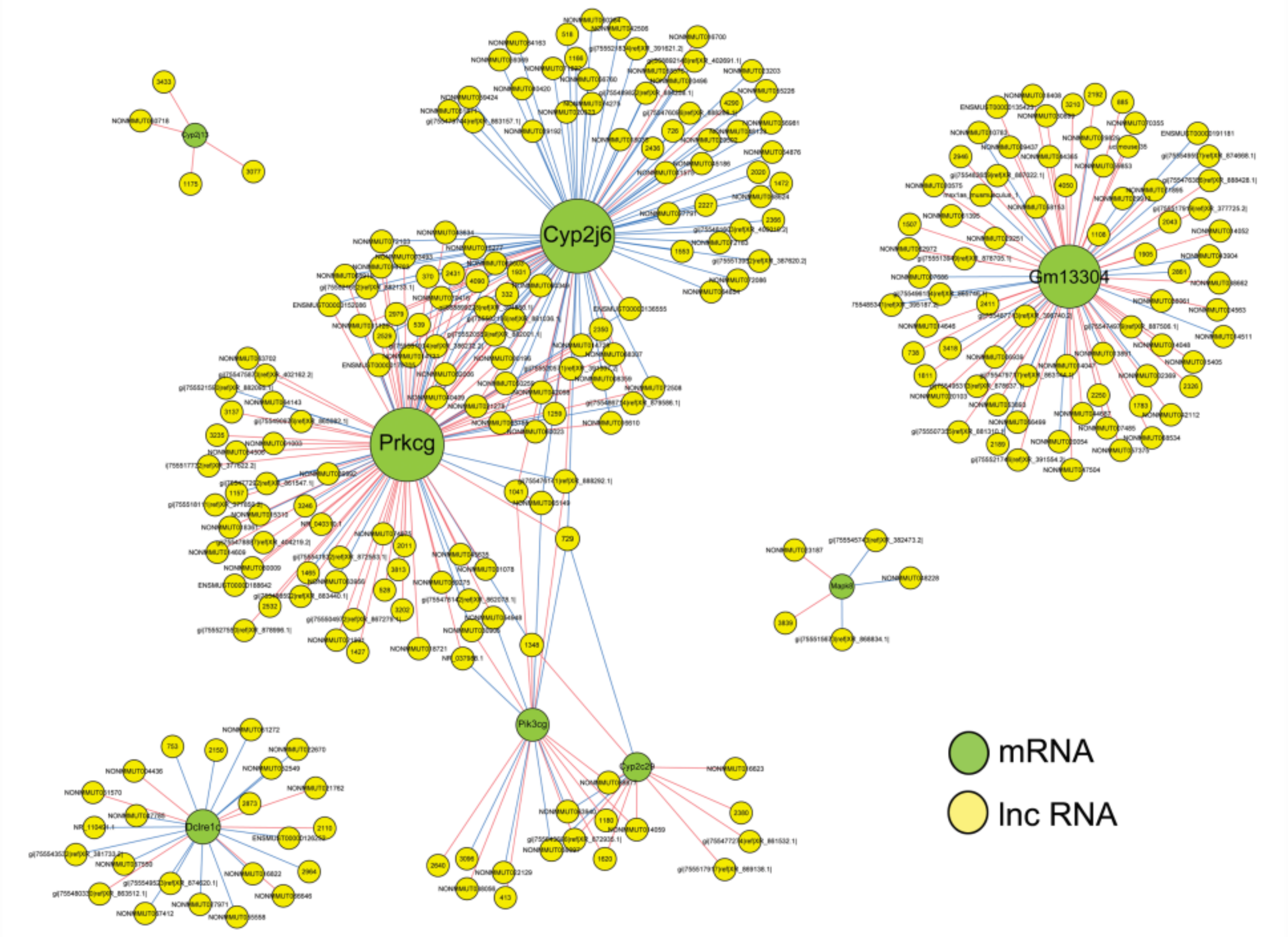

The alteration of lncRNA expression in related to immunity Female+TBI treated female mice

Appendix Table S1.

List of primers used in this paper.

Gene	Primer	Sequence (5′-3′)
**Primers for PCR**		
TFF3	forward	CTGGCTAATGCTGTTGGT
	reverse	TTGTTACACTGCTCCGATG
VEGF	forward	ACATCTTCAAGCCGTCCTGTG
	reverse	GTGCTGTAGGAAGCTCATCTC
MDR1	forward	TACGCCTACTATTACACCG
	reverse	CATCAAACCAGCCTATCTC
Glut1	forward	TATCCTGTTGCCCTTCTGC
	reverse	CCGACCCTCTTCTTTCATCTC
pgk1	forward	GGAAAACCTCCGCTTTCATGTA
	reverse	GCCTTCTGTGGCAGATTCACA
Muc2	forward	CCATTGAGTTTGGGAACATGC
	reverse	TTCGGCTCGGTGTTCAGAG
GAPDH	forward	TGTTTCCTCGTCCCGTAGA
	reverse	CAATCTCCACTTTGCCACTG
**Primers for sequencing**		
515F		GTGCCAGCMGCCGCGGTAA
806R		GGACTACHVGGGTWTCTAAT
