# Supplementary material for: Faecal microbiota transplantation protects against radiation‐induced toxicity
Source: EMBO Mol Med. 2017 Feb 27;9(4):448–61. doi: 10.15252/emmm.201606932 (PMC5376756; doi:10.15252/emmm.201606932)
Supplement: Supplementary file 2 — Expanded View Figures PDF [file EMMM-9-448-s002.pdf]

Expanded View Figures

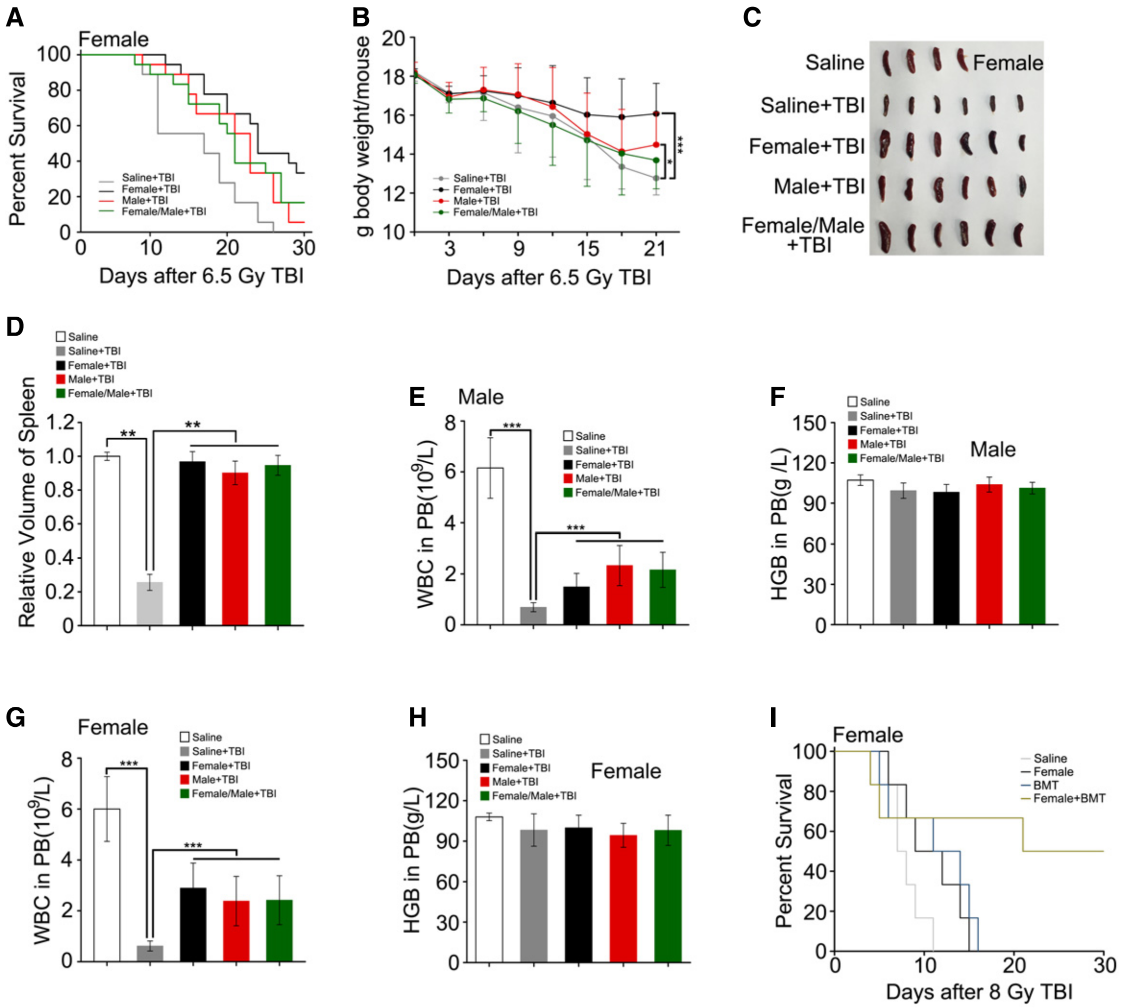

Figure EV1. Gavage of faecal microbiota protects against radiation-induced death and hematopoietic toxicity.

- A Female mice received 10 days of oral gavage with saline, male gut microbes, female gut microbes or male/female gut microbes mixture after 6.5 Gy TBI. Kaplan–Meier survival analysis of the mice was performed. $n = 18$, $P < 0.05$ by log-rank test between saline + TBI group and female + TBI group.
- B Female mice received 10 days of oral gavage with saline, male gut microbes, female gut microbes or male/female gut microbes mixture after 6.5 Gy TBI, the body weight was measured. Significant differences are shown relative to Saline + TBI group: *$P < 0.05$, ***$P < 0.001$; Student's *t*-test, $n = 18$ per group.
- C, D Photographs (C) and volume (D) of dissected spleens from radiation-exposed female mice, the spleens were obtained at day 15 after 6.5 Gy TBI. Mean ± SD. Significant differences are shown relative to Saline + TBI group: **$P < 0.01$; Student's *t*-test, $n = 4$ in Saline group, $n = 6$ in experimental groups.
- E, F WBC and HGB counts in PB were measured in irradiated male mice, the PB were obtained at day 15 after 6.5 Gy TBI. Mean ± SD. Significant differences are shown relative to the saline + TBI group: ***$P < 0.005$; Student's *t*-test, $n = 12$ per group.
- G, H WBC and HGB counts in PB were measured in radiation-exposed female mice, the PB were obtained at day 15 after 6.5 Gy TBI. Mean ± SD. Statistically significant differences are shown relative to the saline + TBI group: ***$P < 0.005$; Student's *t*-test, $n = 12$ per group.
- I Kaplan–Meier survival analysis of the irradiated female mice treated with saline, FMT, BMT or FMT combined with BMT was performed. $P < 0.005$ by log-rank test between FMT combined with BMT and other three groups, $n = 12$ per group.

© 2017 The Authors

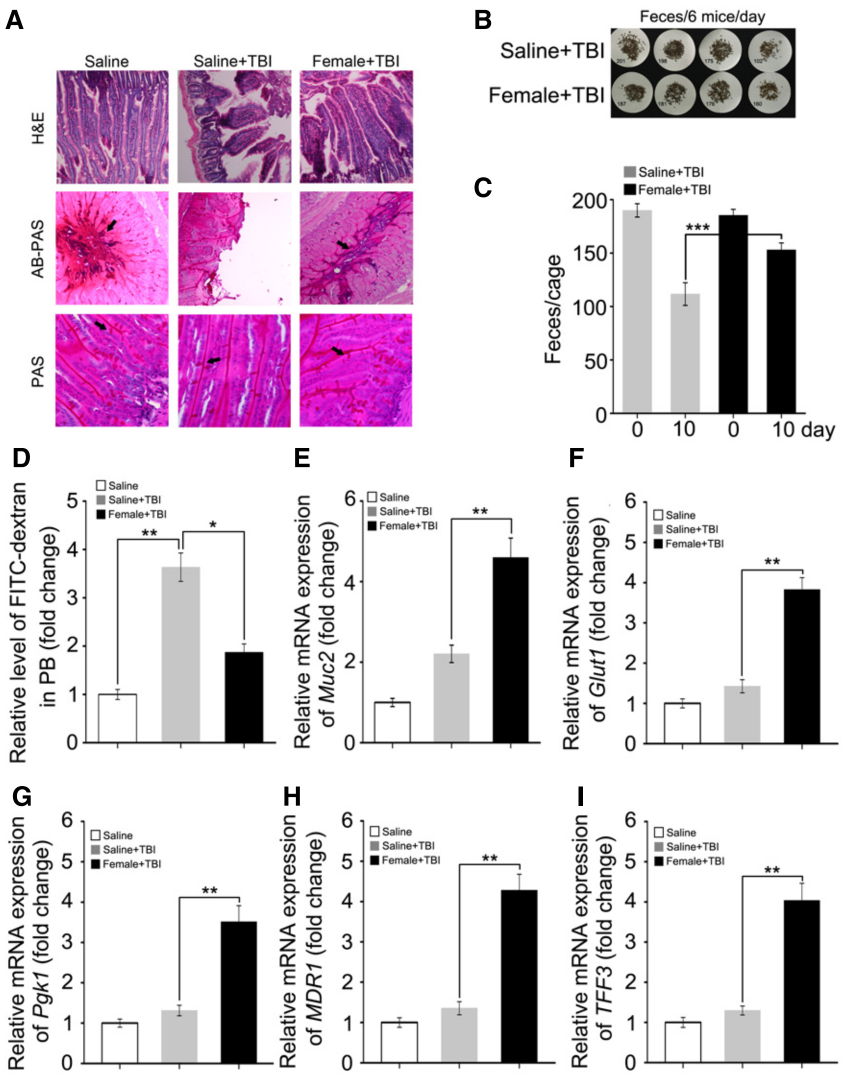

Figure EV2. Faecal microbiota transplant ameliorates GI tract function and epithelial integrity after irradiation.

Female mice were separated into two groups after 6.5 Gy gamma ray exposure, one cohort was treated with saline as control, and the other was treated with sex-matched FMT.

- A The morphology of small intestine in radiation-induced mice treated with saline or sex-matched FMT is shown by H&E, AB-PAS and PAS staining, the small intestine tissues were obtained at day 21 after TBI. The arrows point to the mucus layer or goblet cells.
- B, C Faecal pellet counts removed from cage bedding per 3 day from representative cages. Mean ± SD, $n = 6$ mice per treatment. ***$P < 0.001$ by Student's t-test between Saline + TBI and Female + TBI group.
- D The FITC–dextran in peripheral blood from saline-treated and sex-matched FMT mice was assessed at day 21 after irradiation exposure. Mean ± SD. Significant differences are indicated: *$P < 0.05$, **$P < 0.01$; Student's t-test, $n = 6$ per group.
- E–I The expression levels of *Muc2*, *Glut1*, *Pgk1*, *MDR1* and *TFF3* were examined in saline-treated and sex-matched FMT mice by quantitative PCR, the small intestine tissues were obtained at day 21 after TBI. Mean ± SD. Statistically significant differences are indicated: **$P < 0.01$; Student's t-test, $n = 12$ per group.

 © 2017 The Authors

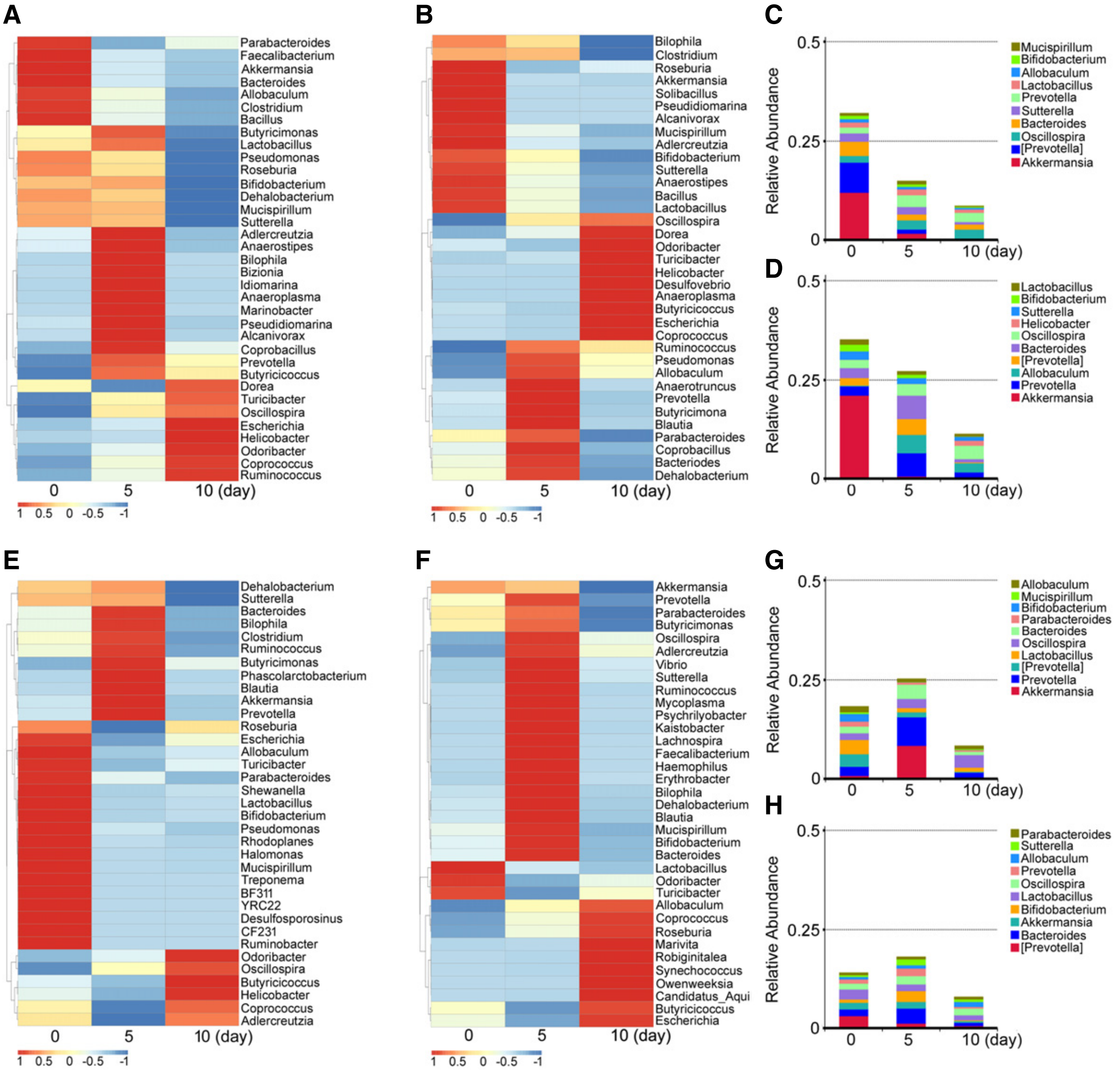

Figure EV3. Faecal microbiota transplantation alters gut bacterial composition profile after irradiation.

A, B The alteration of intestinal bacterial patterns at the genus level in sex-mismatched FMT (A) and sex-mixed FMT (B) male mice was assessed using 16S high-throughput sequencing after irradiation at days 5 and 10, $n = 4$. The heat map is colour-based on row *Z*-scores. The mice with the highest and lowest bacterial level are in red and blue, respectively.

C, D The relative abundance of top 10 bacteria at the genus level in sex-mismatched FMT (C) and sex-mixed FMT (D) male mice was assessed using 16S high-throughput sequencing after irradiation at days 5 and 10, $n = 4$.

E, F The alteration of intestinal bacterial patterns at the genus level in sex-mismatched FMT (E) and sex-mixed FMT (F) female mice was assessed using 16S high-throughput sequencing after irradiation at days 5 and 10, $n = 4$. The heat map is colour-based on row *Z*-scores. The mice with highest and lowest bacterial level are in red and blue, respectively.

G, H The relative abundance of top 10 bacteria at the genus level in sex-mismatched FMT (G) and sex-mixed FMT (H) female mice was assessed using 16S high-throughput sequencing after irradiation at days 5 and 10, $n = 4$.

© 2017 The Authors

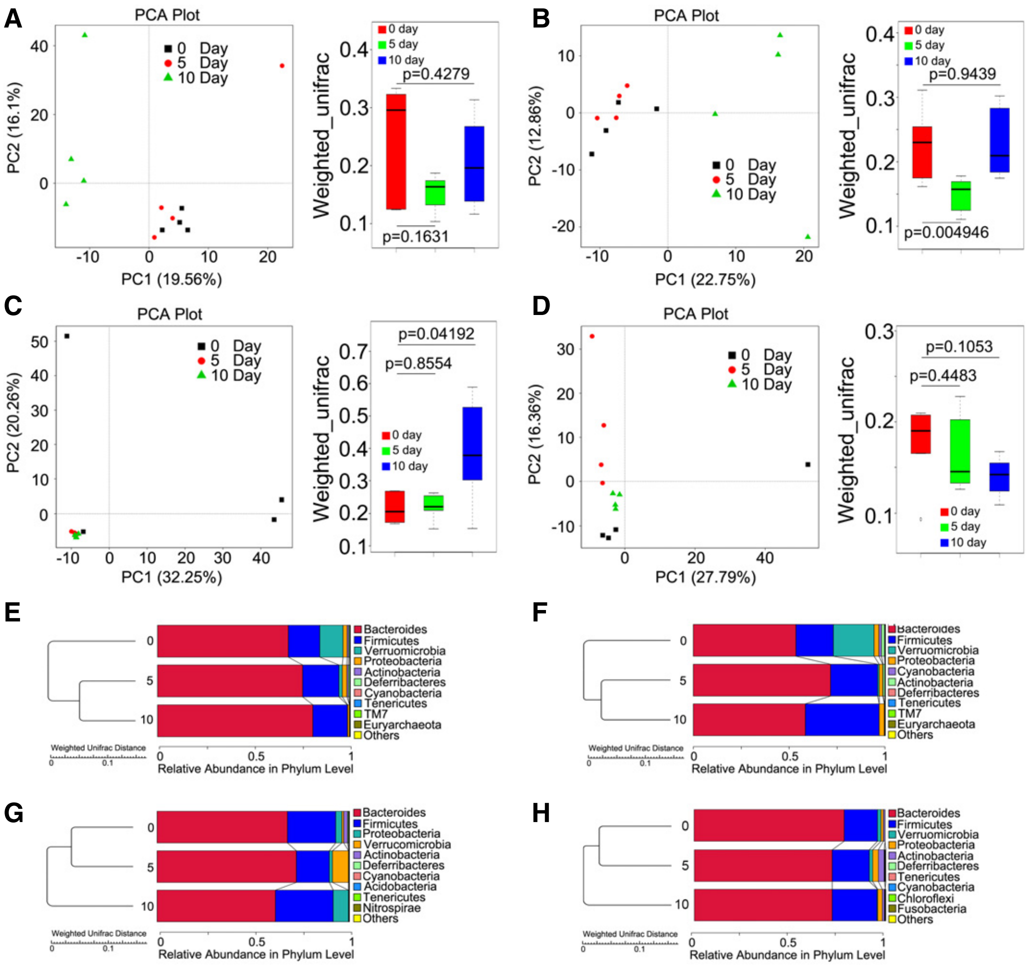

Figure EV4. FMT shifts gut bacterial composition profile after irradiation.

A, B Principal component and β-diversity analysis were used to measure the shift of intestinal bacterial composition profile in sex-mismatched FMT (A) and sex-mixed FMT (B) male mice after irradiation at days 5 and 10, $n = 4$. Statistically significant differences are indicated: Wilcoxon rank sum test. The top and bottom boundaries of each box indicate the 75th and 25th quartile values, respectively, and lines within each box represent the 50th quartile (median) values. Ends of whiskers mark the lowest and highest diversity values in each instance.

C, D Principal component and β-diversity analysis were used to measure the shift of intestinal bacterial composition structure in sex-mismatched FMT (C) and sex-mixed FMT (D) female mice after irradiation at days 5 and 10, $n = 4$. Statistically significant differences are indicated: Wilcoxon rank sum test. The top and bottom boundaries of each box indicate the 75th and 25th quartile values, respectively, and lines within each box represent the 50th quartile (median) values. Ends of whiskers mark the lowest and highest diversity values in each instance.

E, F The relative abundance of enteric bacteria at the phylum level in sex-mismatched FMT (E) and sex-mixed FMT (F) from male mice was assessed using 16S high-throughput sequencing after irradiation at days 5 and 10.

G, H The relative abundance of enteric bacteria at the phylum level in sex-mismatched FMT (G) and sex-mixed FMT (H) from female mice was assessed using 16S high-throughput sequencing after irradiation at days 5 and 10.

 © 2017 The Authors

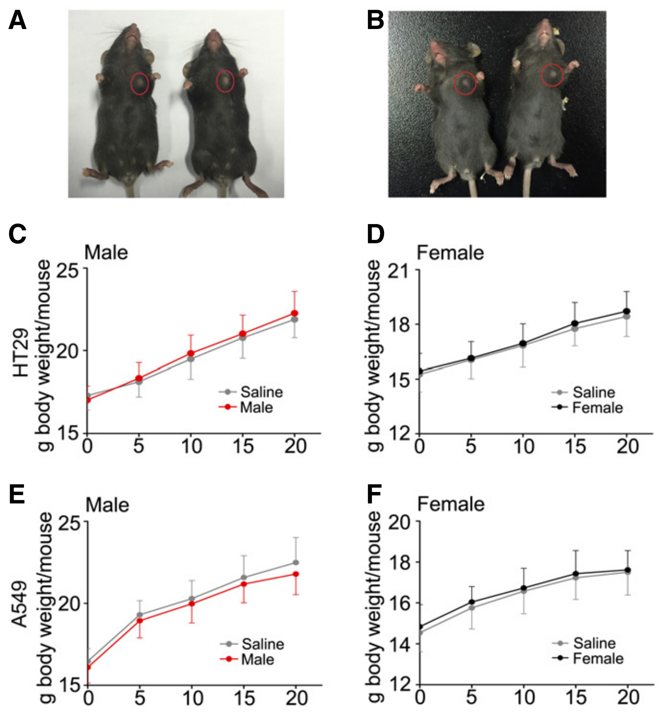

Figure EV5. Gavage of gut microbiota enhances angiogenesis without accelerating tumour growth.

A, B Photographs of tumours transplanted into female (A) and male (B) C57BL/6 mice subcutaneously.

C, D The body weight curve of male (C) and female (D) C57BL/6 mice transplanted with HT29 subcutaneously. Mean ± SD. None significant; Student's *t*-test, $n = 5$ per group.

E, F The body weight curve of male and female C57BL/6 mice transplanted with A549 subcutaneously. Mean ± SD. Statistically significant differences are indicated: Student's *t*-test. None significant; Student's *t*-test, $n = 5$ per group.

© 2017 The Authors
